# Isozygous and selectable marker-free MSTN knockout cloned pigs generated by the combined use of CRISPR/Cas9 and Cre/LoxP

**DOI:** 10.1038/srep31729

**Published:** 2016-08-17

**Authors:** Yanzhen Bi, Zaidong Hua, Ximei Liu, Wenjun Hua, Hongyan Ren, Hongwei Xiao, Liping Zhang, Li Li, Zhirui Wang, Götz Laible, Yan Wang, Faming Dong, Xinmin Zheng

**Affiliations:** 1 Hubei Key Laboratory of Animal Embryo Engineering and Molecular Breeding, Hubei Institute of Animal Science and Veterinary Medicine, Hubei Academy of AgroSciences, Wuhan 430064 China; 2 College of Animal Science, Henan University of Science and Technology, 263 Kaiyuan Avenue, Luoyang, 471023 China; 3 AgResearch, Ruakura Research Centre, Private Bag 3123, Hamilton, New Zealand; 4 Analysis and Testing Center, Institute of Hydrobiology, Chinese Academy of Sciences, Wuhan 430072 China

## Abstract

Predictable, clean genetic modification (GM) in livestock is important for reliable phenotyping and biosafety. Here we reported the generation of isozygous, functional myostatin (MSTN) knockout cloned pigs free of selectable marker gene (SMG) by CRISPR/Cas9 and Cre/LoxP. CRISPR/Cas9-mediated homologous recombination (HR) was exploited to knock out (KO) one allele of MSTN in pig primary cells. Cre recombinase was then used to excise the SMG with an efficiency of 82.7%. The SMG-free non-EGFP cells were isolated by flow cytometery and immediately used as donor nuclei for nuclear transfer. A total of 685 reconstructed embryos were transferred into three surrogates with one delivering two male live piglets. Molecular testing verified the mono-allelic MSTN KO and SMG deletion in these cloned pigs. Western blots showed approximately 50% decrease in MSTN and concurrent increased expression of myogenic genes in muscle. Histological examination revealed the enhanced myofiber quantity but myofiber size remained unaltered. Ultrasonic detection showed the increased longissimus muscle size and decreased backfat thickness. Precision editing of pig MSTN gene has generated isozygous, SMG-free MSTN KO cloned founders, which guaranteed a reliable route for elite livestock production and a strategy to minimize potential biological risks.

Predictable and clean GM in livestock is becoming increasingly important as the introduction of unwanted sequences like SMGs has incurred strong public concerns about the safety for food, biodiversity, and agro ecosystems. SMGs are frequently used in cell-mediated transgenesis to enable the isolation of transgenic cells. But once the manipulation has been achieved, they no longer serve any purpose in the GM livestock generated from such cells. On the contrary, SMGs that remain in livestock could be a liability for the following reasons: (1) the presence of SMGs might interfere with the proper expression of introduced as well as nearby endogenous genes, potentially distorting genotypic and phenotypic analysis and adversely affecting overall performance; (2) the proteins encoded by SMGs may confer host species resistance to antibiotics and thus undermine its therapeutic effects when administered to treat infections; (3) the small number of SMGs that can be used in mammalian cells restricts the opportunities for more complex, multiple-step genetic manipulations; and (4) consumers hold concerns about their biological safety and impacts upon biodiversity and the environment^1^. Therefore, clean GM technology, with its ability to remove SMGs once they are no longer needed, is highly desirable.

There are several methods that can be used to generate SMG-free GM livestock. However, intrinsic technical limitations of these methods have so far hampered the implementation of clean transgenic methodologies as routine practice. For example, direct microinjection of an expression cassette into zygotes avoids the use of any SMG, but it suffers from a very low efficiency, random integration and potential mosacism, rendering this method impractical and expensive. In contrast, delivery of mRNA or protein encoding programmable nucleases like CRISPR/Cas9 has proven to be an efficient strategy for generating SMG-free transgenic livestock. Though, the high efficiencies strictly apply only to the introduction of non-defined, random mutations as a result of non-homologous end joining repair (NHEJ). Moreover, the method’s tendency to create compound mutations and mosaic animals are additional barriers for developing lines of livestock with defined, heritable genotypes^2^,^3^,^4^. Hence, there is still an unmet demand for a more controllable approach that enables the production of GM livestock with precision genome alterations that are free of SMGs.

The Cre/LoxP recombination system is widely used in transgenic mice for tissue-specific gene targeting as well as gene excision. However, the application of Cre/LoxP has yet to be well characterized in GM livestock, and only few studies describe its utility in large animal species. Three reports applied cell-permeable TAT-Cre recombinase or Cre-expressing adenovirus to delete SMG in goat and cattle cells, respectively^5^,^6^,^7^, but these reports did not make a quantitative analysis upon the efficiency of Cre/LoxP-mediated marker excision. Three studies used Cre-expressing plasmid to remove SMG in recloned cattle fibroblasts, but they failed to rule out the possibility of random integration of Cre-expressing plasmid^8^,^9^,^10^. Two groups reported the creation of Cre-expressing pigs for the *in vivo* deletion of SMGs, but this approach was costly and labor-intensive^1^,^11^. Thus, the practical utility of the Cre/LoxP system for the cost-effective and efficient production of SMG-free GM livestock has yet to be demonstrated.

MSTN, also known as growth and differentiation factor 8 (GDF-8), is a member of the transforming growth factor β (TGF-β) superfamily. It is a negative regulator of skeletal muscle development, and its suppression results in enhanced muscle growth and increased leanness of carcass composition. MSTN inactivation is the molecular cause of the double muscling phenotype exhibited in the Belgian Blue and Piedmontese breeds of beef cattle^12^,^13^,^14^. Homozygous MSTN mutant mice were up to 30% larger in body weight and their muscles had larger cross-sectional fiber areas (hypertrophy) as well as a greater number of fibers (hyperplasia) compared to wild-type controls^15^. All these observations suggest that MSTN is an ideal target for increasing muscle growth and improving meat production by genetic manipulation in livestock.

In this report we carried out a two-step process to successively engineer the MSTN locus in primary pig cells. We first applied CRISPR/Cas9-meditaed HR to mono-allelically KO MSTN. In a second step we deleted the floxed SMG from the targeted allele by Cre recombination. The resultant cleanly targeted pig fibroblasts were immediately used as donor nuclei for nuclear transfer, producing two live male cloned piglets. Molecular tests proved the functional disruption of MSTN and the removal of the SMG in the cloned founders. These isozygous, SMG-free MSTN KO pigs exhibited elevated level of myogenic regulatory factors, muscle hyperplasia, overgrown skeleton muscle and decreased fat mass, which duplicated the “double muscling” phenotype. This work provides the basis for the commercial production of GM elite pigs with increased consumers’ confidence of the absence of biological risks.

## Results

### CRISPR/Cas9 mediated efficient HR at the MSTN locus in pig cells

**Figure 1:**
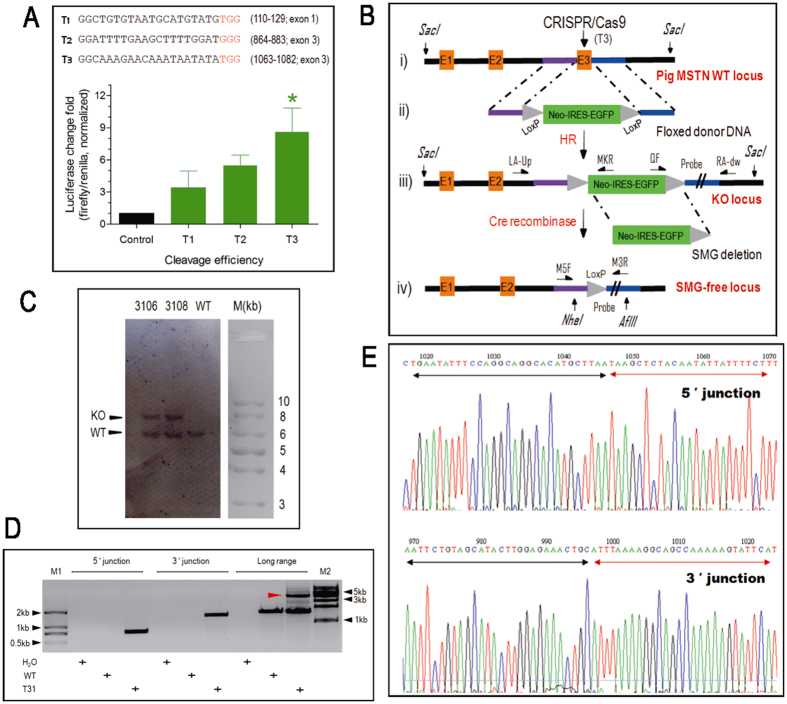
CRISPR/Cas9 mediates efficient and precise HR in both immortal and primary porcine cells. (**A**) Cleavage efficiency of targeting sites within the pig MSTN gene. The three selected target sites for homologous recombination (T1, T2 and T3, shown at the top of the panel) were analyzed using the SSA reporter assay. *, significantly different. Error bar, mean ± SD. (**B**) Strategy for MSTN targeting and SMG deletion. Depicted is i) the schematic outline of the pig MSTN gene, with exons shown in orange and the homology used in the donor DNA in purple (5′ homology arm) and blue (3′ homology arm); ii) schematic map of the donor DNA with homology arms and floxed (LoxP, grey arrow heads) SMG cassette (green rectangle); iii) the correctly targeted locus and iv) the clean targeted locus and the excised SMG following Cre/LoxP-mediated recombination. Primers used for junction and long range PCR are indicated by half arrows. Restriction sites (vertical arrow) and probe (3′ homology arm, blue rectangle with double slash) for Southern blot are shown. (**C**) Validation of MSTN targeting in PK15 cells. For a correct HR event, a 9898 bp fragment was detected in the correctly targeted locus (KO). A 6869 bp fragment was also detected (WT), indicating that the HR was mono-allelic. NC, negative control using the PK15 genomic DNA. The detected fragments migrated slightly faster than corresponding marker because of the high AT-content (AT% = 66.84%) of the MSTN locus. (**D**) Junction and long range PCR for detecting MSTN targeting in pig primary cells (T31 cell clone). 5′and 3′junction PCR produced a 962 bp band and a 1503 bp band, respectively. Long range PCR produced a 1737 bp band for WT allele and a 4773 bp band for KO allele (red arrow). M1 and M2 were DL2000 and 1 kb DNA ladders, respectively. H_2_O, blank control; wt, wild-type genomic DNA. (**E**) Sequencing of the junction PCR products. The top and bottom sequences are for 5′and 3′ junctions, respectively. The fluorescence readout is shown for one of the clones, the others being identical. Black and red arrows stand for the native genomic sequences and the homolog sequences, respectively.

CRISPR/Cas9 recognizes target sites by sequence complementarity to its guide RNA (gRNA) but requires a juxtapositioned PAM sequence (5′-NGG-3′) to induce a double-stranded break (DSB) 3-bp upstream of the PAM motif. To satisfy these requirements, the ZifiT Targeter online tool was used to predict candidate targeting sites in the coding sequence of pig MSTN gene^16^. The candidate sites were further filtered by BLAST against pig genome (UCSC) to exclude those having an identity larger than 50%. Three best target sites (T1 from exon 1, T2 and T3 from exon 3) were selected and engineered into the px300 backbone vector (Addgene) to construct the Cas9/gRNA expression plasmids^17^. The target-specific cleavage activity of the three gRNAs was then evaluated using a single-stranded annealing (SSA) assay^18^. To do this, the targeting regions around each of the targeting sites (up to 300 bp) were cloned into luciferase reporter plasmid pSSA-Luc and co-transfected along with the Cas9/gRNA plasmids into the porcine PK15 cell line. Subsequently SSA showed that while all the three tested CRISPRs (T1-T3) presented target-specific cleavage activity, the Cas9 nuclease complex with the gRNA for T3 had the best cleavage efficiency, with an 8-fold change in luciferase activity being achieved (Fig. 1A). Based on these observations, T3 targeting site was preferred to mutate pig MSTN gene.

One potential issue with the programmable nucleases is the introduction of small arbitrary indels in the targeting sequence. Even in some cases this has led to failure to disrupt the expression of the target gene^2^. This limitation has been circumvented by taking advantage of DSB created by programmable nucleases to greatly enhance the efficiency of HR, thus facilitating a precise and predictable modification^19^. To ensure a precise and functional disruption of the pig MSTN gene, we engineered a floxed donor DNA as repair template, along with CRISPR/Cas9 editor, to introduce predictable mutation in the target site. The donor DNA was designed to delete the MSTN exon 3 (172 bp) which encodes the C-terminal fragment of the mature form of MSTN. It also contained the SMG that was floxed by two LoxP motifs, which allows the excision of SMG by Cre/LoxP recombination. Transformation of the floxed donor DNA into a Cre-expressing *E.coli* strain (BM25.8) demonstrated that Cre recombinase can readily excise the SMG from the donor DNA (Supplementary Fig. 1).

In order to prove the effectiveness of Cas9/gRNA-mediated HR, we next co-electroporated the Cas9/T3 gRNA expression vector along with the floxed donor DNA into PK15 cells. Following selection, 32 EGFP-positive cell clones were genotyped by Southern blot. For two of the cell clones (3106 and 3108) we detected the expected 9898 bp for a correctly targeted MSTN gene, indicating a targeting efficiency of 6.3% (2/32). We also observed the 6869 bp fragment that represented the wild-type allele, indicating that in both cell clones the targeting events were mono-allelic (Fig. 1B,C). Following the full validation of Cas9/gRNA-mediated HR, we then performed MSTN targeting in Hubei white pig primary embryonic fibroblasts. Following selection, we identified 13 EGFP-positive cell clones that were screened by junction and long range PCR for correct targeting events. Only one cell clone (T31) produced the expected amplification fragments consistent with the successful targeting of exon 3 of MSTN (Fig. 1D). Sequencing of the 5′- (962 bp, primers LA-up/MKR) and 3′– (1503 bp, primers QF/RA-dw) junction PCR products confirmed the correct site-specific recombination between the homologous arms of the donor DNA and the endogenous MSTN gene (Fig. 1E). The long range PCR amplification (primers LA-up/RA-dw) of both, the wild type allele (1701 bp) and the targeted allele (4773 bp) fragments showed that the T31 cell clone was heterozygous for the edited allele. The observed targeting efficiency in these primary cells was 7.7% (1/13) which was similar to what we determined in PK15 cells.

### Cre efficiently excises the floxed SMG in the targeted primary cell clone T31

**Figure 2:**
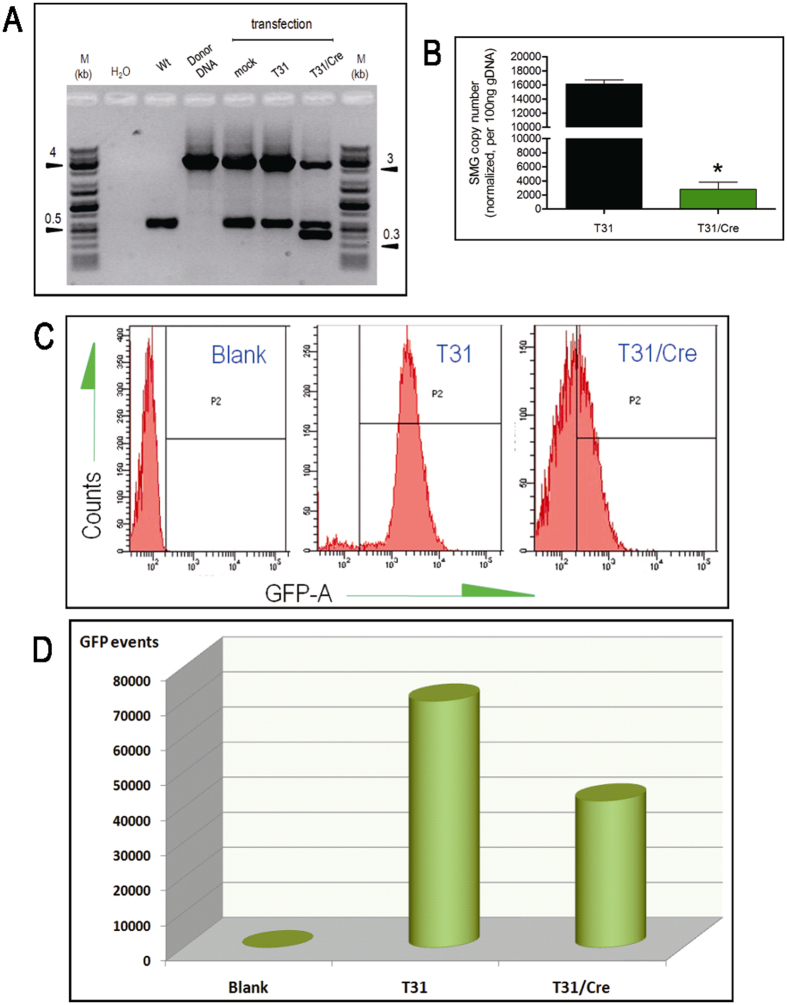
Cre-mediated SMG deletion and isolation of marker-free donor cells. (**A**) Detection of Cre-mediated SMG deletion by end-point PCR. Primer pair M5F and M3R was used to generate a 396 bp band (clean locus), a 534 bp band (WT allele) and a 3357 bp band (KO allele). Mock: non-electroporated T31 cells; T31: T31 cells electroporated with buffer only and T31/Cre: T31 cells electroporated with Cre mRNA. (**B**) Efficiency of SMG deletion. Shown are relative SMG copy numbers determined by real-time PCR in T31 cell electroporated with buffer only (T31, black bar) and T31 cell electroporated with Cre mRNA (T31/Cre, green bar). *, significantly different. Error bar, mean ± SD from technical triplicates. (**C**) Flow cytometery of GFP florescence emitted by wild type porcine embryonic fibroblasts (blank), T31 cells transfected with buffer only (T31) and T31 cells transfected with Cre mRNA (T31/Cre). The results for the different samples are graphically represented as cell numbers (counts) versus GFP average fluorescence (GFP-A). (**D**) Quantification of GFP-positive cells. The amount of GFP-positive cells was quantified from the cell populations of blank, T31 and T31/Cre samples. The non-GFP fluorescing cells from the Cre mRNA-treated T31 cell population (T31/Cre) in this experiment were sorted by flow cytometery and used for SCNT.

For Cre-mediated deletion of the SMG we decided to use Cre mRNA to avoid the risk of unwanted integrations with the use of a Cre expression plasmid. As a diagnostic assay half of the transfected cells were tested by PCR with primers M5F, located upstream of the 5′LoxP motif, and M3R, located downstream of the 3′LoxP motif which can generate three possible fragments indicative of i) the unaltered targeted allele still containing the SMG (3357 bp fragment), ii) the wild type allele (534 bp fragment) and iii) the targeted locus that underwent the intended Cre/LoxP–mediated deletion of the SMG (396 bp fragment). End-point PCR analysis demonstrated the appearance of a novel 396 bp fragment, beside the expected fragments for the wild type and SMG-containing targeted allele, confirming that the SMG cassette had been deleted in a substantial proportion of cells (Fig. 2A). Sequencing of the products by TA cloning confirmed that accurate Cre-mediated removal of the SMG had occurred, with only one LoxP motif remaining at the targeted MSTN locus.

In order to quantify the deletion efficiency, real-time PCR was carried out using primers M3R and UTR-F1, with the latter located within the SMG. This revealed that Cre expression decreased the SMG copy number from 16150 ± 341.2 copies (n = 3) per 100 ng of genomic DNA in untreated cells to 2798 ± 598.2 copies (n = 3) in treated cells, representing a deletion efficiency of 82.7% (Fig. 2B). Analysis of the amplification products of the real-time PCR products further corroborated a large reduction of SMG copy number as a result of Cre expression (Supplementary Fig. 2).

### SMG-free, MSTN KO cells were suitable donor cells for the production of MSTN KO cloned pigs

**Figure 3:**
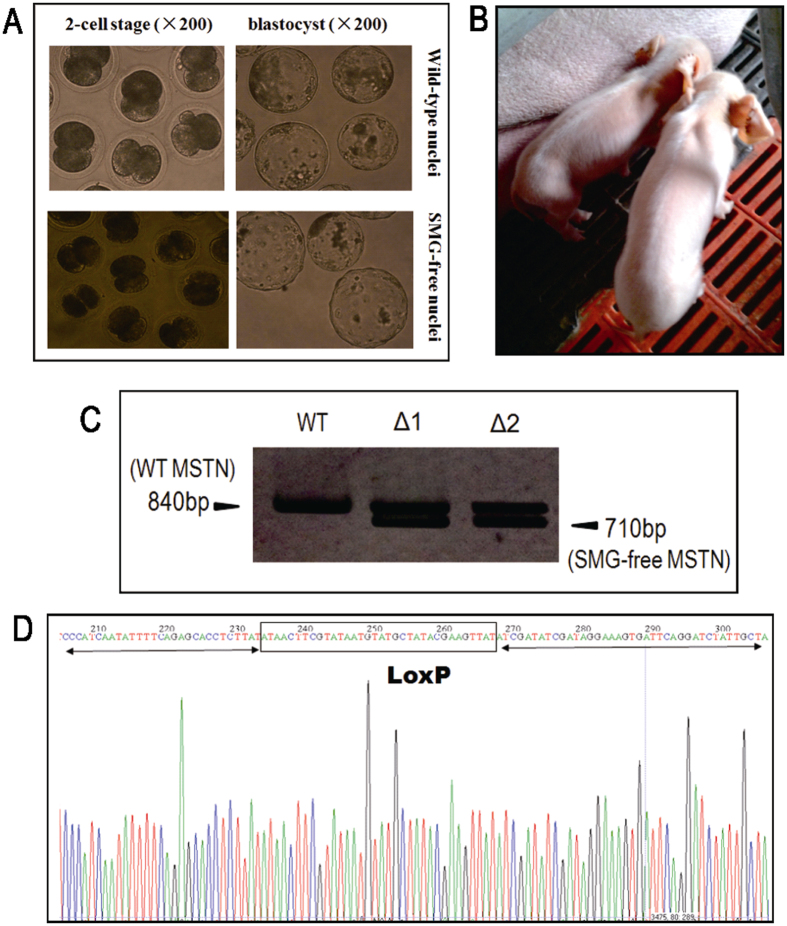
Production and genotyping of SMG-free MSTN KO pigs via SCNT. (**A**) Image (200 × magnification) of SCNT embryos reconstructed using wild type cells (WT) and SMG-free MSTN KO cells as nuclei donors. (**B**) Picture of the two newborn SMG-free MSTN KO piglets produced by SCNT. (**C**) Confirmation of genotype of the cloned SMG-free MSTN KO piglets by Southern blot. WT: wild-type control; Δ1 and Δ2: SMG-free MSTN KO pigs, respectively. (**D**) Sequence of the SMG-free MSTN locus from pig Δ1. The black frame highlights the remaining single LoxP motif and the arrows indicate adjacent endogenous sequences homologous to the 5′ and 3′ arms used in the donor DNA.

After confirming the efficient deletion of the SMG in T31 cell clone by Cre recombinase, we next used fluorescence assisted cell sorting (FACS) to isolate non-EGFP cells, which served as an indirect marker for SMG-free cells, from the other half of the Cre-mRNA treated cells. Flow cytometric analysis showed that the number of EGFP positive cells in the Cre-treated population was reduced by 40.5%, when compared to the control cells (Fig. 2C,D). Sorted, non-fluorescent cells were immediately used without any further culture for generating cloned pigs by somatic cell nuclear transfer (SCNT). No notable difference was observed in the *in vitro* developmental competency of embryos reconstructed with nuclei from wild type and sorted, SMG-free MSTN+/− cells (Fig. 3A; Supplementary Table 1). A total of 685 reconstructed MSTN KO embryos were transferred into 3 recipient sows. While two of the recipients did not get pregnant, the third sow established a pregnancy and delivered two live piglets at full term (Fig. 3B). Genomic DNA was extracted from ear biopsies of the two piglets for genotyping. PCR and Southern blot both demonstrated the mono-allelic disruption of the MSTN locus and absence of the SMG in the two piglets (Fig. 3C,D; Supplementary Fig. 3). Taken together, these results validated our enrichment strategy for SMG-free KO donor cells using FACS and demonstrated the successful generation of live pigs with a clean, mono-allelic MSTN KO from these cells.

### Augmented expression of myogenesis regulators and myofiber hyperplasia in MSTN KO pigs

**Figure 4:**
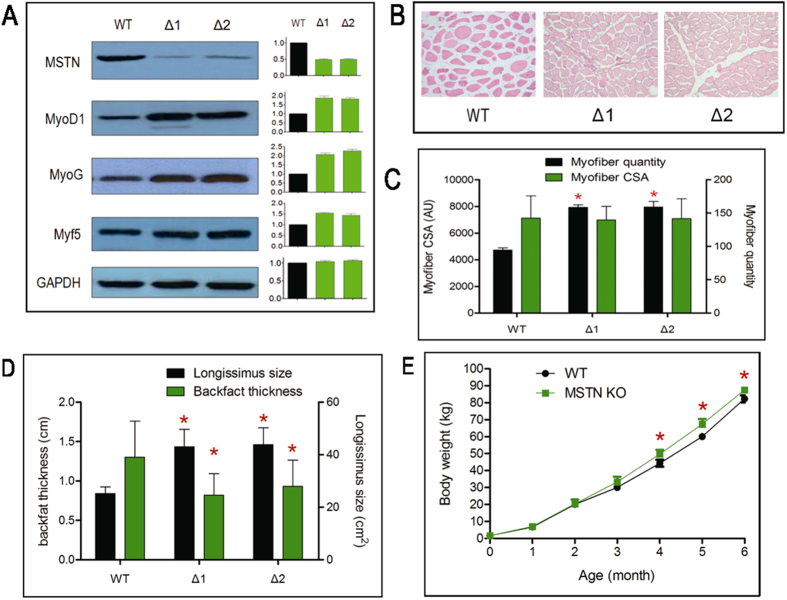
Molecular and phenotypic analysis of the SMG-free MSTN KO cloned pigs. (**A**) Western blot for MSTN and myogenesis-associated proteins. Immunoblots with antibodies specific for the proteins indicated on the left using muscle protein extracts derived from wild type (WT) and the two SMG-free MSTN KO piglets (Δ1, Δ2). Relative quantification of the detected protein levels (WT = 1.0) is shown as bar graphs (WT, black; MSTN KO, green) on the right. MSTN; myoststin; MyoD1: myoblast determination protein 1; MyoG: myogenin; Myf5: myogenic factor 5; GAPDH: glyceraldehyde-3-phosphate dehydrogenase, serving the internal control for equal loading amount. Error bar, mean ± SD. (**B**) Representative images (100× magnification) of stained muscle sections from wild type (WT) and SMG-free MSTN KO piglets (Δ1, Δ2) to compare quantity and size of myofiber in an identical unit area. (**C**) Quantification of the number and size of myofibers. The quantity of myofibers is shown as black bars (average of 10 random sections) and the size of the myofibers was measured in arbitrary units (AU) of the cross section areas (CSA) of 100 myofibers (green bars). *, statistically significant. Error bar, mean ± SD. (**D**) The longissimus muscle size (black bars) and backfat thickness (green bars) of wild type (WT) pigs compared to SMG-free MSTN KO pigs (Δ1, Δ2). Shown are the mean values of measurements at three different spots along the muscle with error bars representing mean ± SD. *, statistically significant. (**E**) Growth curve of the SMG-free MSTN KO and wild type (WT) pigs from birth to 6-month old. Shown are the average growth curves for the two KO piglets (green) and wild type controls (black; n = 3). *, statistically significant. Error bar, mean ± SD.

Next we examined the MSTN protein expression by western blot using muscle biopsies taken from the mono-allelic MSTN knockout cloned pigs at 6-month old. As shown in Fig. 4A, MSTN expression dropped by approximately 50% in the two cloned pigs compared to a wild-type control. The decrease of the MSTN protein level was consistent with approximately 50% reduction of MSTN mRNA abundance in the MSTN KO pigs compared to the wild-type control as determined by quantitative PCR (Supplementary Fig. 4). MSTN is a negative regulator of muscle growth which inhibits myoblast proliferation and differentiation by inactivating downstream target genes involved in myogenesis^20^,^21^. We therefore examined the effect of the reduced MSTN level on the expression of three myogenic transcription factors, including myoblast determination protein 1 (MyoD1), myogenin (MyoG) and myogenic factor 5 (MYF5) in the two pigs. Consistent with the inhibitory role of MSTN, western blot demonstrated the augmented expression of these three important myogenic factors (Fig. 4A). Histological analysis of muscle sections revealed that the number of myofiber per unit area in the two MSTN KO piglets was significantly greater than for the wild-type (94.60 ± 1.056 for WT, 158.5 ± 1.267 for Δ1, 159.2 ± 2.649 for Δ2; *P* < 0.05), while the size of the myofiber remained unaltered (Fig. 4B,C). This indicated that the MSTN KO induced muscle hyperplasia but not hypertrophy in the cloned pigs.

### Skeleton muscle overgrowth and decreased backfat thickness in MSTN KO pigs

The results above prompted us to examine these pigs for phenotypic effects of reduced MSTN levels, namely enhanced skeletal muscle growth and reduced fat accumulation. Using ultrasonic detection, we measured the longissimus size and backfat thickness when the cloned pigs were 6-month old. As shown in Fig. 4D, the longissimus muscle size of MSTN knockout pigs was significantly larger (43.01 ± 4.724 cm^2^ in Δ1, 43.77 ± 4.562 cm^2^ in Δ2, 25.23 ± 1.766 cm^2^ in controls) than in the breeding-, age- and sex-match wild type controls (n = 3), indicating the enhanced skeleton muscle development. The backfat thickness of the MSTN KO pig was significantly thinner (0.769 ± 0.143 cm in Δ1, 0.830 ± 0.136 cm in Δ2, 1.801 ± 0.177 cm in controls) than the controls (n = 3), indicating the decreased adipogenesis due to MSTN KO. The body weight was insignificantly different in the first 3 months, but the MSTN KO pigs were significantly heavier than controls by 12.01%, 12.22% and 11.51%, respectively, between 4-months and 6-months of age (*P* < 0.05) (Fig. 4E). These data were consistent with reported phenotypic changes in MSTN KO mice and provide evidence that we have generated a functional MSTN KO in Hubei white pigs resulting in potential improved carcass characteristics^22^.

### Biosafety risk analysis of the cloned MSTN KO pigs

We performed two different assays to analyze the biosafety risk of the SMG-free MSTN KO cloned pigs. Firstly, primers against Cas9 coding sequence, eukaryotic neomycin resistance gene and prokaryotic ampicilin resistance gene were used for end-point PCR. This test ruled out the possibility of random integration of the Cas9/gRNA or donor DNA sequences (Supplementary Fig. 5). Secondly, biochemical indices were examined in the blood of the two 6-month old mutant and WT pigs. We observed a sharp increment for alkaline phosphatase (ALP) (WT, 6.3 ± 0.94 U/L, n = 3; Δ1, 132.6 U/L; Δ2, 111.10 U/L) and uric acid (UA) (WT, 0, n = 3; Δ1, 37.50 μmol/L; Δ2, 40.80 μmol/L). Total bilirubin (TBIL) was lower in the two MSTN KO pigs (0.1 μmol/L in Δ1 and 0.2 μmol/L in Δ2) than in the wild type pigs (3.9 ± 2.28 μmol/L, n = 3). It was also noteworthy that low density lipid (LDL) were higher in wild type pigs (1.48 ± 0.15 mmol/L, n = 3) compared the MSTN KO pigs (0.80 mmol/L in Δ1 and 0.43 mmol/L in Δ2). No significant difference was detected in all other tested indices in the mutant and WT groups (Supplementary Table 3). The MSTN KO pigs fed, grew and developed indistinguishably from the wild-type pigs.

## Discussion

Marker-free GM livestock signifies an important facet to address public concern on food safety and would help boost consumers’ acceptance to embrace GM products. Several recent studies have reported the generation of marker-free livestock by either microinjection of mRNA or protein of nucleases or somatic cell nuclear transfer using mutant cell clones free of SMGs. However, these methods have only limited predictability as they rely on aberrant DNA repair to generate stochastic indels, not providing the desired predictability and precision of introducing specific, pre-defined mutations. For example, some GM livestock produced with the introduction of zinc finger nucleases (ZFN), transcription activator-like effector nucleases (TALEn) or CRISPR/Cas9 into zygote or primary cells were found to be mosaic and contain multiple different mutations with many of these mutations not resulting in the intended functional disruption of the target genes^2^,^3^,^4^,^23^,^24^,^25^. In this study we demonstrated the site-specific and mutation-specific modification at the pig MSTN gene to generate the isozygous, marker-free MSTN KO cloned pigs. This was achieved by the combined use of CRISPR/Cas9-mediated HR to knock out exon 3 of MSTN and Cre/LoxP recombination to remove the floxed SMG subsequently. Compared to NHEJ-induced mutations and direct zygote microinjection, our cell-mediated method was more predictable and compatible with multistep modifications, allowing the introduction of pre-defined mutations for the intended functional inactivation of the target genes. These isozygous founders makes breeding easier as it excludes the problem with segregating mutant alleles that arise from mosaic mutations.

Cre/LoxP site-specific recombination system has been widely used in transgenic plants and mice for tissue-specific expression of the target genes thanks to its faithful and efficient recombination ability, but it has been less characterized in farm animals. Our study and a recent report demonstrated the potentiality of Cre/LoxP in engineering livestock. Garrels *et al.* took advantage of Cre/LoxP-mediated cassette exchange to generate syngeneic cohort of pigs carrying different reporter transposons at an identical chromosomal location with a comparable efficiency as we showed in this study^26^. It is safe to say that Cre/LoxP technology still has very important applications in genetically modified livestock owning to its highly efficient re-targeting capability like site-specific excision and cassette exchange. In this regard, Cre/LoxP to some extent is not likely to be replaced by other programmed nucleases because of its well defined catalytic kinetics and predictable recombination outcome.

The efficiency of HR is very low in mammalian cells, which has severely limited the application of traditional gene targeting by HR in somatic livestock cells^27^. Until recently, enhanced HR efficiency was achieved using programmable nucleases to create DSB, which greatly facilitates HR^28^,^29^. Several studies used homologous single-stranded oligodeoxyonucleotide (ssODN) as repair template to introduce HR with variable efficiency in different species^30^,^31^, In particular, two groups reported using this technology to obtain an HR efficiency at the MSTN locus in pig somatic cells of 3.64% and 4.4%^32^,^33^, respectively. In this study we observed an even better HR efficiency when targeting MSTN locus in both of immortal (6.3%) and primary (7.7%) porcine cells. Please note that 5′and 3′ homologous arms in the donor DNA used in this work were respectively 750 bp and 800 bp in length, which were much shorter than traditional targeting plasmid^34^,^35^. We reasoned that the differences in HR efficiency might result from targeting a different site in the pig MSTN gene, or use of a different pig breed. Overall, our work demonstrated that CRISPR/Cas9 is a potent system to generate DSB and trigger HR to greatly enhance gene targeting efficiency. This strategy is highly flexible and readily expandable to other porcine genes or other livestock to achieve effective and precision genomic engineering.

Somatic cell nuclear transfer using GM donor cells has been an effective way to generate GM livestock. Two studies have demonstrated that the donor cells that had undergone sequential modifications without the need for rejuvenation by recloning remained competent to produce marker-free cloned cattle either disrupting alpha1, 3-galactosyltransferase or expressing human β-defensin-3 in milk. However, these investigators encountered the low efficiency of isolating marker-free cells or failure to delete the visual marker EGFP from the cloned animals^6^,^36^. In contrast, we employed a reverse strategy to isolate non-EGFP population after Cre/LoxP-catalyzed excision of the SMG. We observed a marked difference between SMG excision from the MSTN KO cells as measured by real-time PCR (82.7%) and the more indirect assessment by loss of GFP –positive cells (40.5% reduction). This was most likely a reflection of the delayed EGFP clearance from cells where the SMG had been excised due to the long half life of EGFP^37^. The reconstructed cloned embryos derived from these marker-free donor cells were developmentally competent *in vitro* and comparable to wide type cells. However, the *in vivo* cloning efficiency for the generation of live piglets was 2/685 = 0.3%, quite low compared to what we had observed in a previous study^38^. This might reflect the differences in intrinsic constraints and requirements for the *in vitro* and *in vivo* development of reconstructed embryos, for which the mechanisms are still largely unknown. It would require a better understanding of the basis of reprogramming in order to improve SCNT efficiency through a rational approach.

MSTN was discovered in a screening of a mouse muscle cDNA library for novel TGF-βfamily members and later studies proved that Its inactivation resulted in the “double muscling” phenotype in mice, sheep, dog, cattle, rabbit and human^39^,^40^,^41^,^42^,^43^,^44^. MSTN null mice exhibit heavy muscle mass resulting from both myofiber hyperplasia and hypertrophy^22^,^45^. In this study, we presented evidence that myofiber hyperplasia but not hypertrophy contributed to the increased skeleton muscle growth in the MSTN KO pigs, which was consistent with three recent reports^32^,^33^,^46^. We reasoned that MSTN might exert its effect on myogenesis in distinct mechanism in different species. We further identified the concomitantly elevated level of myogenic regulatory factors including MyoD1, MyoG and Myf5 as a consequence of the functional loss of MSTN in the pigs. This is consistent with earlier reports that MyoD1 is up-regulated in the absence of MSTN in C2C12 fibroblast cells or MSTN knockout mice^20^,^21^. It was also reported that MSTN is part of a regulatory circuit and forms a close inter-relationship with MyoD1 and IFRD1 in mice^47^. In fact the exact mechanism by which MSTN modulates myogenesis is still not well-understood, even leading to contradicting results^48^,^49^. Our findings provided the possibility to decipher the precise impact of MSTN on muscle formation in pigs, which thus may elicit comparative explanations on its distinct roles in muscle growth physiology among species.

In summary, we have successfully generated non-mosaic and marker-free pigs with a predictable and precise KO of MSTN combining CRISPR/Cas9 and Cre/LoxP technologies. MSTN was disrupted mono-allelically in these pigs, resulting in an over-expression of myogenesis-associated genes. Histological analysis revealed an increase in myofiber quantity, while the myofiber size remained unaltered. Phenotypic tests demonstrated enhanced skeletal muscle growth and reduced backfat thickness in the MSTN KO pigs. Our work takes advantage of predictable and clean gene targeting method to prevent the occurrence of inhomogenecity that results from NHEJ-induced mutations. Furthermore, the deletion of the SMG minimizes associated potential biosafety and environmental risks and may help to encourage wider acceptance. This work will provide valuable new insights into the benefits of clean, precisely modified livestock and their respective safety for human food consumption.

## Methods

### Animal ethnics

All experimental procedures involving animals were reviewed, approved, supervised and conducted in accordance with the Guidance of Use and Care of Laboratory Animals and Livestock from the Animal Care Committee of Institute of Animal Science and Veterinary Medicine, Hubei Academy of AgroSciences. All the wild-type and MSTN KO pigs were raised with same diet and identical conditions.

### Plasmids and strains

CRISPR/Cas9/gRNA expression plasmid px330 was obtained from Addgene. pIRES2-EGFP, pUC19, pcDNA3.1(+), Cre recombinase expression plasmid pTurbo-Cre (with 5′ nuclear localization signal), SSA reporter plasmid pSSA-Luc, targeting plasmid backbone HRX, DH5 α and BM25.8 (Cre-expressing) *E. coli* strains were sourced by Key Laboratory of Animal Embryo Engineering and Molecular Breeding of Hubei Province. TA cloning plasmid was purchased from Takara. Competent *E.coli* DH5α cells were prepared according to standard protocols. A PureLink^®^ HiPure Plasmid Filter Purification Kits were used for midi and maxi preparation of all plasmid DNA (Invitrogen). A nanophotometer P-class was used to measure the quality and quantity of DNA (Implen, Germany). The neomycin selection marker gene (Neo^R^) was subcloned into pIRES2-EGFP by NheI and BamHI to create the CMV-Neo^R^-IRES-EGFP selectable marker cassette, which was subsequently subcloned into pUC19 by SalI and BglII, resulting in a targeting plasmid backbone named HRX. Homologous arms were cloned into HRX by EcoRI/ClaI and SalI/AflII, respectively, resulting in the donor DNA named ΔMSTN. For the SSA reporter assay, the 196bp targeting region at T1 site and 260 bp targeting region at T2 and T3 sites were cloned into pSSA-Luc to generate reporter plasmid named pSSA-Luc-T1 and pSSA-Luc-T2/3.

### DNA manipulation

Pig genomic DNA of cultured cells and tissues was extracted by a Purelink^®^ Genomic DNA Mini kit (Invitrogen). The 804 bp 3′homolog arm was used as the probe and labeled by a DIG High Prime DNA Labeling and Detection Starter Kit I (Roche) in consistence with the manufacture’s manual book. For Southern blot, 20 μg genomic DNA was digested by SacI or NheI and AflII and fractionized in 1% or 2% agarose gel at 20 V overnight. The gel was soaked in alkali solution (0.5 N NaOH) for 2 × 15 minutes and the denatured DNA was transferred to a nylon membrane by the sandwich method. The DNA was fixed by baking at 120 °C for 30 minutes. Hybridization was carried out at 42 °C overnight. The membrane was washed by 2 × SSC/0.5%SDS washing buffer to remove the non-specific binding probes. Color detection was performed with NBT/BCIP solution included in the kit.

### RNA manipulation

The DNA sequence encoding Cre recombinase was amplified by T7-containing primers and its mRNA was synthesized *in vitro* using mMESSAGE mMACHINE mRNA transcription synthesis kit (Life Technologies). Cre mRNA was purified using phenol:chloroform extraction and isopropanol precipitation and eluted in RNase-free water. Total RNA was extracted with Trizol (Invitrogen Inc, USA) in accordance with the manufacturer’s instructions. The mRNAs were then reverse-transcribed with M-MLV (Promega) using oligo-dT primers in a 50 μl reaction system, and the PCR was performed in a 20 μl volume. The pig GAPDH gene was used as an internal control.

### Cell culture

Primary embryonic fibroblast cells of Hubei white pig was isolated from pig fetuses on day 35 of gestation. The heads and internal organs were removed using iris scissors and forceps. The remnants were washed three times with DPBS and the carcass was minced with a surgical blade on a 100-mm culture dish. The minced fetal tissues were dissociated in Dulbecco modified Eagle medium (DMEM, Gibco) supplemented with 0.25% (w/v) trypsin-EDTA (Life Technologies) at 39 °C for 1 to 2 h. Trypsinized cells were washed once by centrifugation at 1000 rpm for 5 min and subsequently seeded into 100-mm plastic culture dishes. Seeded cells were cultured for 6 to 8 days in DMEM supplemented with 10% (v/v) fetal bovine serum (FBS; Hyclone) and 10 μg/ml of penicillin-streptomycin solution (Sigma-Aldrich Corp.) at 39 °C in a humidified atmosphere of 5% CO_2_ and 95% air. After removal of unattached clumps of cells or explants, attached cells were further cultured until confluent, and then passaged and frozen in DMEM containing 20% FBS and 10% dimethylsulfoxide for future use. Electroporation was carried out in a BTX ECM2001 electro cell manipulator. 4 μg plasmid DNA (2 μg Cas9/gRNA plasmid and 2 μg floxed donor DNA) or 5 μg Cre mRNA was used for every 10^6^ cells in a cuvette with 1 mm diameter (voltage 170 v, duration 3 ms, and pulse 1). The electroporation buffer contained 120 mM KCl, 0.15 m CaCl2, 25 mM Hepes, 2 mM EDTA, 5 mM MgCl2, and 10 mM K_2_HPO_4_, with a final pH value 7.6. 800 μg/ml G418 was added to the cells 48 hours post electroporation for selection for 14 days and the single cell clones were isolated by cloning disc and seeded into 48-well plate in duplicates for PCR screening and expansion. The targeted cell clone was then seeded in a 6-well plate to grow into 90% confluence. FACS analysis was performed in BD FACS Aria III. Excitation and emission wavelength for GFP were 488 nm and 520 nm, respectively. Briefly, 5 × 10^5^ cells were electroporated by Cre mRNA, split in duplicates and incubated for 12 hours to recover. After that serum-starving medium (0.5% FBS in DMEM) was used for additional 3 days for simultaneous SMG deletion and cell starvation. One well of the Cre-treated cells was used for test of SMG deletion and the other well used for flow cytometery. The cells were filtered by 100-well net and resuspended in DPBS with a total cell amount of 8 × 10^4^ cells in each sample for sorting. Average GFP fluorescence (GFP-A) was measured and non-EGFP cell population was collected for nuclear transfer.

### SSA reporter assay

PK15 cell line was transfected using Lipofectamine 3000 reagent (Invitrogen) when it reached 70% confluency for SSA reporter assay. Briefly, 2 μg each of Cas9/gRNA expression plasmids for T1, T2 and T3 tagregting sites were co-transfected along with 0.5 μg each of SSA reporter plasmid pSSA-Luc-T1 and pSSA-Luc-T2/3 into PK15 cells with three replicates in parallel, respectively. An internal control plasmid pRL-TK that expresses Renilla luciferase was included in all transfections for data normalization. 48 hours later the cells were lysed in 1 × passive lysis buffer (Promega) and the luciferase activity was measured in a Glomax luminometer (Promega) using a DLR^TM^ Assay (Promega) according to manufacturer’s instruction.

### Pig oocyte *in vitro* maturation and SCNT

Pig ovaries were collected from an abattoir affiliated with COFCO (China Oil and Food Import and Export Corporation) in Wuhan and transported to the laboratory in 0.9% saline (w/v) supplemented with100 IU/ml penicillin G and 100 IU/ml streptomycin sulfate at 25–30 °C. Cumulus-Oocyte complexes(COCs) were collected from follicles with a diameter of 3–8 mm in a collection medium consisting of Medium 199 (Sigma) supplemented with 10%(v/v) pig follicular fluid, 0.1% (w/v) polyvinyl alcohol, 3.05 mM glucose, 0.91 mM sodium pyruvate, 0.57 mM L-Cysteine, 100 IU/ml streptomycin sulphate (GIBCO), 100 IU/ml potassium penicillin G(GIBCO), 10 IU/ml PMSG (Ningbo Second Hormone Factory) and 10 IU/ml hCG (Ningbo Second Hormone Factory). The COCs were incubated for 6d in a 5-well dish under an atmosphere of 5% CO_2_, 95% air and 100% humidity at 39 °C. Polar body I (pb I) of *in vitro* maturated oocyte was aspirated by an Φ 20 μm pipette after 42–44 hrs in TCM 199 maturation medium. The enucleated oocytes and donor cells were fused by a single DC pulse of 1.5 kV/cm for 30 μsec using a BTX Electro-Cell Manipulator 2001 (BTX Inc., San Diego, CA). After 24–48 hrs culture in NCSU-23 medium^50^, the reconstructed embryos were transferred into the oviduct of surrogates (Hubei white pigs sourced from JinYing Animal Science Company Ltd., Wuhan, China). For *in vitro* development, The cloned embryos after activation treatment were washed five times with NCSU-23 containing 4 mg/ml BSA, then cultured in the same medium which had been previously covered paraffin oil in a polystyrene culture dish and equilibrated at 38.5 °C in an atmosphere of 5% CO_2_ in air. The rate of cleavage and blastocyst formation was assessed on day 2 and 7, respectively.

### End point and real-time PCR

LA Taq (Takara) DNA polymerase was used in the end-point PCR testing. A typical LA Taq PCR reaction mixture contained 5 μl 10 × LA PCR buffer (Mg^++^ plus), 8 μl dNTP mixture (2.5 m each), LA Taq polymerase 0.5 μl (5 units/μl), forward and reverse primer 1 μl (20 μM, respectively), template 0.5 μg and PCR-grade water added to 50 μl in total. Cycling condition is 94 °C 2 min, 30 cycles of 94 °C 30s, 55 °C 30s and 72 °C 1 kb/min, followed by final extension of 5 min at 72 °C. 1/5 volume of the PCR product was fractionized by either 1% or 2% agarose gel and photographed by ChemDOC^TM^ XRS + (Biorad). SYBR Green I real-time PCR master mix from Toyobo was used in all quantitative PCR tests in a Rotor Gene6000 real-time rotary analyzer (Corbette Lifescience, Eppendorf). Briefly, the 20 μl reaction mixture included 10 μl 2 × master mix, 0.5 μl primer mix (5 μM each), 1 μl template DNA (100 ng for genomic DNA or 50 ng for plasmid DNA) and 8.5 μl PCR-grade water. A two-step amplification protocol was used with the following parameters: 95 °C for 2 minutes to pre-denature the template and activate Taq DNA polymerase followed by 40 cycles each of denaturation at 95 °C for 8 seconds, annealing and extension at 60 °C for 25 seconds. A final melting temperature analysis from 50 °C to 99 °C was used to ensure amplicon uniformity. Fluorescence was acquired at the step of annealing and extension. All PCR amplifications were performed in triplicate for each treatment. Relative signal intensities were calculated by theΔΔCt method with the built-in software RG6000 series 1.7. Copy number of the SMG cassette was calculated using the single-copy transferrin receptor protein 1 gene (TFRC) as internal control with the following formula: SMG copy number/pig genome = molecules of SMG/molecules of TFRC × 2. For the quantitative expression analysis, cDNAs were assessed using the ΔΔCt method and normalized against the endogenous housekeeping genes GAPDH.

### Western blot

Muscle biopsies were washed by ice-cold DPBS (Hyclone) thrice and cut into small pieces. The muscle pieces were lysed in RIPA buffer (Invitrogen) and sonicated for 5 minutes at a power of about 160 watts (in rounds of 10 seconds sonication/10 seconds rest for each cycle). Keep the sample on ice during the sonication. The sonicated tissues were centrifuged at 10,000 × g for 20 minutes at 4 °C, and then the supernatant was transferred to a fresh microfuge tube. The cultured cells were washed thrice with ice-cold DPBS to remove residual medium as much as possible. Afterwards the cells were lysed by NP40 Cell Lysis Buffer (Invitrogen) containing phenylmethylsulfonyl fluoride (PMSF) with a final concentration of 1 mM. After centrifugation, the supernatants were collected into fresh tubes. Protein concentration of the lysate was measured by Pierce BCA protein assay kit (Thermo Scientific). A total of 25 μg proteins were denatured in boiling water for 5 minutes and chilled in ice.15% SDS-PAGE was used to size-fractionate the proteins. Then they were electrophoretically transferred onto polyvinylidene difluoride membranes (Amersham Biosciences, Piscataway, NJ). After blocking with 2% milk, the membranes were incubated with primary antibodies at 4 °C for 1 hour. The rabbit polyclonal primary antibodies against MSTN (19142-1-AP), MyoG (19249-1-AP), MyoD1 (18943-1-AP) and Myf5 (19705-1-AP) were purchased from ProteinTech Co. Ltd. (Wuhan, China). The monoclonal primary antibody against GAPDH (G8795) was purchased from Sigma-Aldrich. After washing with PBST, the membranes were incubated with rabbit anti-mouse horseradish peroxidase-conjugated secondary antibodies (Sigma-Aldrich) for 2 h at room temperature. The signals were visualized by enhanced chemiluminescence kit (Perkin-Elmer, Norwalk, CT). The western blot assay was performed twice independently, and the densimetric intensity was scanned and calculated by Image J software.

### Ultrasound measurement

Longissimus muscle size and backfat thickness were measured when the pigs were 6-month old at three spots of 4th-5th rib, 10th-11th rib and last rib by Yawei 9000V ultrasonic detector according to the manufacture’s manual book (Yawei Livestock Company Limited, Guangzhou, China).

### Histological examination

Pigs were anesthesized by ear intravenous injection of 25 mg/kg pentobarbital sodium and the skeletal muscle biopsies were surgically taken from the quadriceps femoris. The aliquots of the muscle biopsies were either fixed by 4% paraformaldehyde for hematoxylin and eosin staining or immediately immersed into liquid nitrogen for storage. Paraffin sections of the quadriceps femoris were prepared and stained with hematoxylin and eosin mainly according to previously described procedures^31^. The myofiber quantity in unit area (0.025 mm^2^) was counted in 10 randomly sliced sections, and its average value was shown. For myofiber cross-section area (CSA), 100 myofibers were randomly selected and measured by Image J in term of arbitrary unit (AU).

### Blood biochemistry

Ear venous bloods were collected from the two MSTN KO pigs and three wild-type pigs in the morning after an overnight fast to minimize inter-individual variations due to metabolic effects. Haparin sodium salt-containing vacuum blood tubes (Kangjian Medical Apparatus Co. Ltd., Jiangsu Province, China) were used to prevent coagulation. Blood was centrifuged at 100 × g for 5 minutes in a desktop centrifuge (Sigma) and plasma were isolated and examined for the biochemical indices in a ProCyte Dx^TM^ hematology analyzer (IDEXX Laboratories, Inc., Westbrook, Maine, USA).

### Statistical analysis

Statistical comparisons upon cleaving efficiency in SSA reporter assay, SMG copy number variation, densimetric values in Western blots, myofiber quantity and size, carcass character, body weight, mRNA abundance and blood indices among MSTN KO and WT pigs were determined using the student’s t-test in Prism 4 (GraphPad Software, San Diego California, USA). P < 0.05 was considered as statistically significant.

## Additional Information

**How to cite this article**: Bi, Y. *et al.* Isozygous and selectable marker-free MSTN knockout cloned pigs generated by the combined use of CRISPR/Cas9 and Cre/LoxP. *Sci. Rep.* **6**, 31729; doi: 10.1038/srep31729 (2016).

## Supplementary Material

Supplementary Information
