# Supplementary material for: Isozygous and selectable marker-free MSTN knockout cloned pigs generated by the combined use of CRISPR/Cas9 and Cre/LoxP
Source: Sci Rep. 2016 Aug 17;6:31729. doi: 10.1038/srep31729 (PMC4987667; doi:10.1038/srep31729)
Supplement: Supplementary Information [file srep31729-s1.pdf]

SUPPLEMENTARY INFORMATION

Isozygous and selectable marker-free MSTN knockout cloned pigs generated by the combined use of CRISPR/Cas9 and Cre/LoxP

Yanzhen Bi[1,*], Zaidong Hua[1,*], Ximei Liu[1,*], Wenjun Hua[1], Hongyan Ren[1], Hongwei Xiao[1], Liping Zhang[1], Li Li[1], Zhirui Wang[2], Götz Laible[3], Yan Wang[4], Faming Dong[2,**], Xinmin Zheng[1,**]

[1]Hubei Key Laboratory of Animal Embryo Engineering and Molecular Breeding, Hubei Institute of Animal Science and Veterinary Medicine, Hubei Academy of AgroSciences, Wuhan 430064 China.

[2]College of Animal Science, Henan University of Science and Technology, 263 Kaiyuan Avenue, Luoyang, 471023 China.

[3] AgResearch, Ruakura Research Centre, Private Bag 3123, Hamilton, New Zealand.

[4]Analysis and Testing Center, Institute of Hydrobiology, Chinese Academy of Sciences, Wuhan 430072 China.

* These authors made equal contribution to this work.

** Corresponding author: Prof. Xinmin Zheng, email, novorigin@126.com; Prof. Faming Dong, email, dfming24@aliyun.com

Supplementary Figure S1. Functionality of floxed donor DNA in Cre-expressing BM25.8 *E.coli* strain

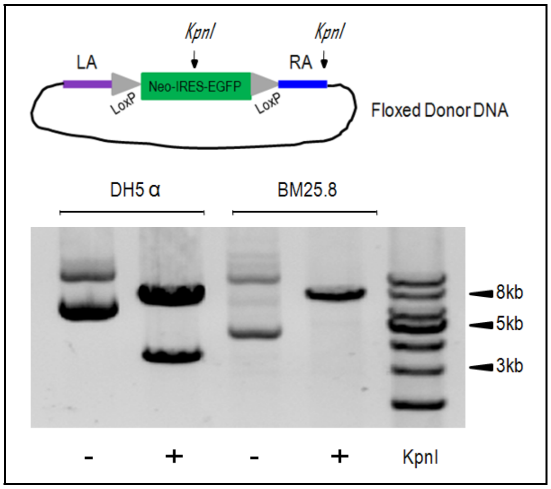

The schematic map of the floxed donor DNA was shown on the top. SMG (Neo^R-IRES-EGFP) was flanked by two LoxP motifs. The floxed donor DNA was transformed into either DH5α or Cre-expressing BM25.8 *E.coli* strains. The resultant plasmids were digested by *KpnI* at 37℃ overnight. Cre recombinase expressed in BM25.8 triggered the recombination, resulting in the deletion of SMG. Therefore only one band was observed, while two bands were generated from the plasmids produced by DH5α control strain.

Supplementary Figure S2. SMG copy number alteration by agarose gel electrophoresis

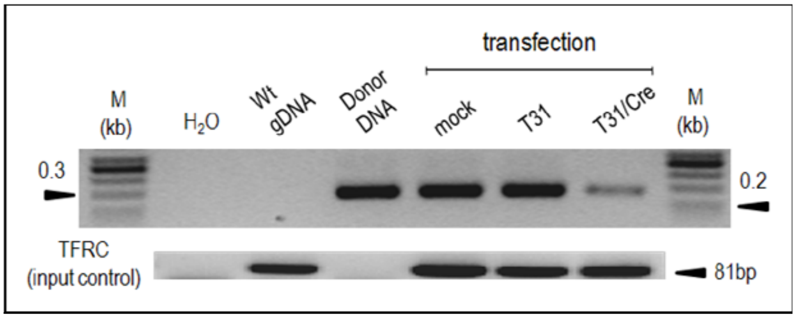

Products from the real-time PCR as indicated in Fig. 2B were fractionized by agarose gel to outline the SMG copy number alteration due to the Cre/LoxP-catalyzed recombination. TFRC gene was used as the internal control.

Supplementary Figure S3. PCR genotyping of the SMG-free MSTN KO cloned pigs

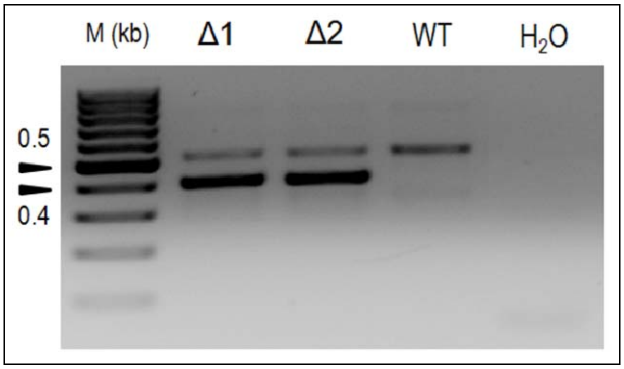

End-point PCR was performed with primer pair M5F and M3R flanking the floxed SMG cassette using genomic DNA isolated from the two cloned piglets (Δ1, Δ2) and a wild type control (WT). H_2O is the blank control. A novel 396bp band appeared in the two piglets, indicating that the SMG had been deleted from the knockout allele.

Supplementary Figure S4. MSTN mRNA abundance in the WT and mutant pigs.

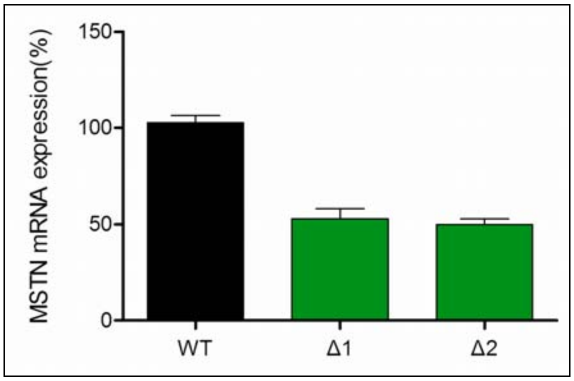

MSTN mRNA abundance was quantified by $\Delta\Delta$Ct method and normalized to GAPDH. Error bar represents the standard deviation (SD).

Supplementary Figure S5. End-point PCR analysis of potential random integration of Cas9/gRNA plasmid or donor DNA.

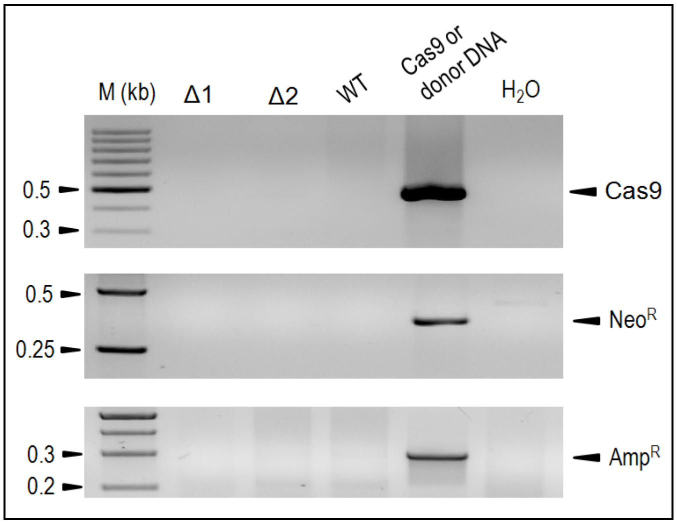

Cas9, Neo^R and Amp^R sequences were amplified by PCR from genomic DNA of the mutant or WT pigs and size-fractionized by agarose gel electrophoresis.

Supplementary Table S1. *In vitro* **developmental competency of reconstructed embryo produced from wild-type and SMG-free nuclei**

Nuclei type	No. reconstructed embryos	Cleavage rate/%	Blastocyst rate/%
Wild-type	138	76.09 (105/138)	10.48 (11/105)
SMG-free	121	74.38 (90/121)	9.92 (12/121)

We compared the developmental competency of reconstructed embryo produced from wild-type and SMG-free nuclei to see if the passages and genomic manipulations would exert adverse effect on embryo quality. The cleavage rate and blastocyst rate were not significantly different between the two types of cloned embryos.

Supplementary Table S2. Primers and oligos used in this study

Primer	Sequence（5'-3')	Comments
NeoF	CTAGCTAGCATGATTGAACAAGATGGATTGC (NheI)	For NeoR CDS to be cloned into pIRES2-EGFP plasmid, creating CMV-NeoR-IRES-EGFP selectable marker cassette
NeoR	CGCGGATCCCGTGCGTTTTATTCTGTCTTTTTATT (BamHI)	
BFZF	TCCGTCGACTAGTTATTAATAGTAATCAATTACG (SalI)	To subclone CMV-NeoR-IRES-EGFP into pUC19 plasmid
BFZR	ATCAGATCTGCAGTGAAAAAAATGCTTTATTTG (BglII)	
LA-F	gcagGAATTCCCTAGTGAATGGAGGAAGG (EcoRI)	To clone 5'homologous arm of pig MSTN, 758bp in length. LoxP site is underlined
LA-R	gcagATCGATATAACTTCGTATAGCATACATTATACGAAGTTATTTATTTGTTCTTTGCCAT (ClaI)	
RA-F	cagcGTCGACATAACTTCGTATAATGTATGCTATACGAAGTTATATATGGGAAAATTCCAGCC (SalI)	To clone 3'homologous arm of pig MSTN, 804bp in length. LoxP site is underlined. This fragment was also used for Southern blot.
RA-R	cacgatgcatGCAGTTTCTCCAAGTATGC (AflII)	
LA-Up	CTACCACTCCCTTCATCACCTAC	5' junction PCR primers, 962bp in length
MKR	GGGCTATGAACTAATGACCCCG	
EGFP-QF1	TGAACCGCATCGAGCTGAAGGG	3' junction PCR primers, 1500bp in length.
RA-Down	CTGCAGTGTTGCAAAGGCAGGC	
M5F	CACCTAGTGAATGGAGGAAGGATGAG	To prove the deletion activity
M3R	AGTTAGAGGGTAACGACAGCATCG	
UTR-F1	GGTTACAAATAAAGCAATAGC	In combination with M3R to quantify the copy number of transgene.
TFRC-QF	GAGACAGAAACTTTCGAAGC	The internal control for quantification of SMG copy number, 81bp in length
TFRC-QR	GAAGTCTGTGGTATCCAATCC	
SSA-T1F	ATCAGATCTAGAAATAAGAACAAGGAGAAAG (BglII)	To clone T1 target site into pSSA-Luc reporter plasmid, 196bp in length
SSA-T1R	AAGCTCGAGTTGAAGATTTAGTGTTTTGTCTCC (XhoI)	
RV-M	GAGCGGATAACAATTTCACACAGG	Sequencing primer in TA cloning
SSA-T3F	ATCAGATCTTCGTTACCCTCTAACTGTGG (BglII)	To clone T2/3 target site into pSSA-Luc reporter plasmid, 260bp in length
SSA-T3R	AAGCTCGAGCTACCATGGCTGGAATTTTCCC (XhoI)	
Cre-T7	GCGTAATACGACTCACTATAGGG ATGGCACCC AAGAAGAAGA	Cre mRNA *in vitro* transcription
Cre-pA	GATCTCCATAAGAGAAGAGGGACA	
hSpCas9-F1	GGTGCAGACCTACAACCAGC	Detection of Cas9 DNA sequence

hSpCas9-R1	AGAGCTTTCAGCAGGGTCAG	
Neo-F1	AGAGGCTATTCGGCTATGAC	Detection of neomycin resistance gene
Neo-R1	TCGCCGCCAAGCTCTTCAGC	
Ori-F3	CGGTGTTGGGTCGTTTGTTC	Detection of ampicillin resistance gene
Ori-R3	ACTACGGCTACACTAGAAGG	
HMP1	GCACCCAAAAGATATAAGGCCA	Quantification of MSTN mRNA abundance, 138bp in length
HMP2	CATCTTTGTGGGAGTACAGCA	
GAPDH-F	ACCCAGAAGACTGTGGATGG	Internal control in real-time PCR, 125bp in length
GAPDH-R	TTGAGCTCAGGGATGACCTT	

Supplementary Table S3. Hematological and biochemical indices of the 6-month old mutant and WT pigs

Indices	WT (n=3)	Δ1	Δ2
Alanine aminotransferase (ALT, U/L)	37.87±2.80	81	33.90
Aspartate aminotransferase (AST, U/L)	76.93±31.34	38.4	59.10
**Alkaline phosphatase (ALP, U/L)**	**6.3±0.94**	**132.6**	**111.10**
Gltamyltranspeptidase (GGT, U/L)	137.93±12.64	82.40	158.60
**Total bilirubin (TBIL, μmol/L)**	**3.9±2.28**	**0.1**	**0.2**
Total protein (TP, g/L)	68.87±4.91	73.5	77.20
Albumin (ALB, g/L)	34.03±1.39	39.20	36.40
Globulin (GLB, g/L)	34.83±3.52	34.30	49.30
Glucose (GLU, mmol/L)	4.68±0.65	3.45	3.32
Triglyceride (TG, mmol/L)	0.34±0.08	0.26	0.22
Serum total cholesterol (TC, mmol/L)	2.20±0.24	1.94	1.03
High-density lipoprotein (HDL, mmol/L)	0.74±0.08	0.96	0.58
**Low density lipoprotein (LDL, mmol/L)**	**1.48±0.15**	**0.80**	**0.43**
**Uric Acid (UA, μmol/L)**	**0**	**37.50**	**40.80**
Carbamide (Urea, mmol/L)	4.97±1.44	6.69	3.96
Serum creatinine (Crea, μmol/L)	118.27±16.42	49.50	80.70
Lactic dehydrogenase (LDH, U/L)	986.2±576.28	733.20	752.70
α-hydroxybutyric dehydrogenase (α-HBDH, U/L)	941.1±400.83	646.20	580.90

Data are mean±SD in WT group. ALP and UA were increased in MSTN KO pigs compared to WT controls. TBIL and LDL were decreased in MSTN KO pigs compared to WT controls.

There was no much difference among other indices in the two groups.
